# Vitrification of human blastocysts for couples undergoing assisted reproduction: an updated review

**DOI:** 10.3389/fcell.2024.1398049

**Published:** 2024-05-17

**Authors:** Romualdo Sciorio, Luca Tramontano, Gerard Campos, Pier Francesco Greco, Giuseppe Mondrone, Anna Surbone, Ermanno Greco, Riccardo Talevi, Nicola Pluchino, Steven Fleming

**Affiliations:** ^1^ Fertility Medicine and Gynaecological Endocrinology Unit, Department Woman Mother Child, Lausanne University Hospital, Lausanne, Switzerland; ^2^ Département de Gynécologie-Obstétrique, Réseau Hospitalier Neuchâtelois, Neuchâtel, Switzerland; ^3^ Fertility Geisinger Medical Center, Women’s Health Fertility Clinic, Danville, PA, United States; ^4^ GIREXX Fertility Clinics, Girona-Barcelona, Spain; ^5^ Villa Mafalda, Centre for Reproductive Medicine, Rome, Italy; ^6^ IVF Research, Education, Development S.R.L., Caserta, Italy; ^7^ Department of Obstetrics and Gynecology, UniCamillus, International Medical University, Rome, Italy; ^8^ Dipartimento di Biologia Strutturale e Funzionale, Universita’ di Napoli ‘Federico II’, Complesso Universitario di Monte S, Napoli, Italy; ^9^ Discipline of Anatomy and Histology, School of Medical Sciences, University of Sydney, Sydney, NSW, Australia

**Keywords:** assisted reproductive technology, infertility, slow-freezing, vitrification, human blastocysts, clinical pregnancy outcomes

## Abstract

Over the past 40 years there has been a worldwide critical change in the field of assisted reproduction technology (ART), leading to the increased application of single blastocyst transfer, which is extremely important to avoid the risks of multiple pregnancy and associated complications for both mother and babies. Indeed, advancements in ART over the last few decades have been obtained thanks to several improvements, including ovarian stimulation, embryo culture conditions and, of course, progress in cryopreservation methods, especially with the application of vitrification. The ability to cryopreserve human embryos has improved significantly with vitrification compared to the initially adopted slow-freezing procedures. Since the introduction of vitrification, it has become the gold standard method to effectively cryopreserve human blastocysts. However, some new protocols are now being explored, such as the short warming procedure and even shorter exposure to the equilibration solution before vitrification, which seem to provide optimal results. Therefore, the main aim of the current narrative review, will be to illustrate the benefit of vitrification as an effective method to cryopreserve the human blastocyst and to illustrate new protocols and variations which in future may increase the performance of vitrification protocols.

## Introduction

In recent times the expectation of pregnancy after a relationship or marriage has often met with infertility challenges as many couples nowadays have not been able to conceive at all or to carry a pregnancy to term. Increasing numbers of couples have difficulties conceiving and as a result this might induce anxiety, sadness, depression, and sometimes might even be the cause of divorce. Since the birth of L. J. Brown in 1978 (Steptoe and Edwards, 1978), assisted reproductive technology (ART) has been steadily on the rise, allowing millions of infertile couples to conceive (Calhaz-Jorge et al., 2020). Currently, it has been estimated that more than eight million children have been born globally following ART (Smeenk et al., 2023). Further, infertility affects millions of people around the world who are of reproductive age; it has been reported that between 48 million couples and 186 million people worldwide suffer from infertility (Smeenk et al., 2023). The advancement in ART has been achieved thanks to many improvements, including ovarian stimulation protocols, sperm preparation techniques, fertilization, and embryo culture methods and, importantly, progress in cryopreservation of gametes and embryos. Cryopreservation has improved cumulative pregnancy and live birth outcomes and has enabled the application of a single embryo transfer (ET) policy, with a reduction in the risk of multiple gestation. Cryopreservation of human embryos by “slow freezing” procedures started in the 1980s (Chen, 1986), which was subsequently replaced by the “vitrification” procedure (Kuwayama et al., 2005). This practice has been considered a real breakthrough in ART, allowing embryologists to obtain a higher survival rate at warming and, following frozen ET (FET), increased pregnancy outcomes compared to the slow freezing procedure (Potdar et al., 2014; Rienzi et al., 2017). Practically, cryopreservation enables the long-term preservation of cells and tissues (gametes/embryos) at ultra-low temperatures in a state of suspended animation. To obtain that state it is fundamental to avoid ice crystal formation, which will irreversibly damage cell membranes and induce cell death. This can be achieved by vitrification, using a high concentration of cryoprotective agents (CPAs) to increase viscosity and inhibit the growth and formation of ice crystals, finally inducing the vitrification solution to enter a “glassy state” (Huber et al., 2014). One of the most applied CPAs during cryopreservation of human gametes and embryos is dimethyl sulfoxide (DMSO): an amphipathic chemical compound. Especially when used at high concentration, DMSO might impact cellular functions, metabolism, enzyme activities, cell growth and apoptosis (Santos et al., 2003; Iwatani et al., 2006; Verheijen et al., 2019) and, as suggested by animal studies, might induce epigenetic dysregulation (Kohaya et al., 2013; Christou-Kent et al., 2020). Further studies have reported that vitrification might be associated with increased levels of reactive oxygen species (ROS) and apoptotic events (Kohaya et al., 2013; Zhao et al., 2016; Verheijen et al., 2019). Importantly, ART procedures and cryopreservation coincide with the period of epigenome reprogramming, going from fertilization through to blastocyst formation. Modifications at this very delicate point might have repercussions during the future life of the conceived baby (Sendzikaite and Kelsey, 2019; Barberet et al., 2020; Chen et al., 2022). Thus, elements associated with vitrification protocols, such as the consequences of chilling, osmotic induced stress, and high concentrations of CPAs, might have an influence on epigenome integrity and transcript stability, with potential consequences for the offspring (Barberet et al., 2020; Chen et al., 2022). Therefore, the main aim of this narrative review is to describe the value and efficacy of the vitrification programme in modern ART, and highlight the current state of knowledge about the impact that a high concentration of CPAs, used with the vitrification process, might have on epigenetic alteration and possible consequences for future generations.

## History of cryopreservation

Cryopreservation protocols enable freezing of biological materials with subsequent storage in liquid nitrogen (LN_2_; −196°C), to interrupt all biological processes and maintain their viability for future use. The frozen embryos can be easily warmed and replaced into the uterus, without any ovarian stimulation, for couples who want an additional pregnancy or should fresh embryo replacement prove unsuccessful. Since the 1970s, cryopreservation in ART cycles has been successfully utilized to freeze gametes and embryos. The first report of a live birth following the transfer of a cryopreserved-thawed embryo was announced in Australia by Trounson and Mohr in 1983 using the so-called “slow freezing” procedure (Trounson and Mohr, 1983). In the late 1990s, a great advancement in the field was achieved with the introduction of the “vitrification” protocol in Japan and Australia (Mukaida and Wanda, 1998; Kuwayama et al., 2005). Rapidly, the vitrification method replaced slow-freezing and was applied to achieve better outcomes in terms of cryo-survival and pregnancy rates, compared to standard freezing. Indeed, the ability to cryopreserve human embryos, using vitrification, has notably improved and, currently, there is enough evidence showing that results obtained from vitrification are enhanced compared to those achieved with the slow freezing protocols (Li et al., 2014; Sciorio et al., 2018; Sciorio et al., 2019). The success of vitrification is correlated with several features, such as the temperature during the vitrification and warming steps, which partly depends upon the choice of the carrier applied and, most importantly, the concentration and type of CPAs used. Regarding temperature, it has been clearly demonstrated in the literature that the warming rate is just as important as the cooling rate, if not more so. Seki and Mazur reported that cryo-damage might also be induced by re-crystallization during the warming step (Seki and Mazur, 2009). They examined the relationship between cooling *versus* warming rates in a mouse oocyte model and concluded that a warming rate of at least 3,000°C/min was imperative to obtain an acceptable survival rate above 80%. As mentioned earlier, selection of the carrier applied, and whether LN_2_ encounters the droplet containing the embryo (open vitrification) or not (closed vitrification) might impact the cooling rate and impair the efficiency of the vitrification process. Additionally, using an open device for vitrification, the LN_2_ itself can contain microbes or pathogens, and therefore, concerns have been raised over sterility which might be compromised during the process, increasing the risk of potential cross-contamination between the embryo being vitrified and the LN_2_. This risk, though minimal, is however not completely excluded, and has been described by others (Bielanski et al., 2000; Bielanski, 2012). Published studies have shown that closed vitrification devices can be used for successful cryopreservation of human embryos (Vanderzwalmen et al., 2010; Chen et al., 2013; Sciorio et al., 2019) but closed carriers are still not totally accepted by the IVF community owing to the perception that they might reduce the survival rate. Studies have suggested the benefits of sterilization of LN_2_ using ultraviolet light (Parmegiani et al., 2010) or storage in the vapour phase of LN_2_, which contains a lower density of contaminants (Cobo and Romero, 2010).

## Cryoprotectants used with vitrification: advantages and concerns

The choice and the concentration of CPAs represent an important decision to take, which is directly associated with vitrification outcomes. CPAs are supplemented to the equilibration and vitrification media to defend cells from cryo-damage (Table 1). There are two different types of CPAs: “penetrating” and “non-penetrating”. The former have a small molecular weight (less than 400 Da) and are therefore able to pass through the cell membrane and, once inside, protect the cell from cryo-induced damage. This group includes glycerol, ethylene glycol (EG), DMSO, propylene glycol or 1,2 propanediol (PROH) and acetamide. Probably DMSO, glycerol and PROH are the more common and mostly used penetrating CPAs. Non-penetrating CPAs are non-diffusible, normally have a higher molecular weight, and therefore cannot cross the cell membrane. Examples are trehalose, sucrose, glucose, mannitol, galactose, polyethylene glycol and polyvinylpyrrolidone, since they have a high molecular weight, and are therefore able to induce an osmotic gradient which diffuses water from inside to outside the cell, thereby reducing the risk of intracellular ice formation (Karlsson and Toner, 1996). Indeed, it is worth mentioning that CPAs, especially when used at high concentrations, might cause toxicity in a time and temperature dependent manner (Fuller, 2004). With this concern, a few studies have been recently published, reporting some detrimental effects of the cryopreservation procedure on the epigenetic makeup of the embryo (Barberet et al., 2020; Chen et al., 2022). Also, reports suggest that cryopreservation may affect some cellular processes, such as cell functionality, protein expression, DNA integrity, cytoskeletal and nuclear structures (Kader et al., 2009; Kopeika et al., 2015; Verheijen et al., 2019; Palomares and Rodriguez, 2022). As cryopreservation becomes more widely used, not only in ART but also in other fields, such as regenerative medicine or transplantation medicine, it is extremely important to examine potential genomic and epigenetic changes associated with current cryopreservation practices (Xu et al., 2010; Chen et al., 2022). A retrospective study reported that singleton pregnancies obtained from vitrified-warmed embryo replacement are associated with less obstetric and perinatal morbidity, and had reduced odds of low birth weight, preterm birth and small for gestational age (SGA) (Pelkonen and Koivunen, 2010; Wennerholm and Henningson, 2013; Ishihara et al., 2014; Maheshwari et al., 2018). Added to this, there is an increased need to optimize the vitrification procedure, and probably embryo exposure times to CPAs could be slightly modified. An attempt to investigate this concern has been performed by Xiong and colleagues in 517 frozen-warmed human embryos (Xiong et al., 2016). They split FET cycles into four groups according to the equilibration time pre-vitrification: 5–6 min, 7–8 min, 9–10 min and 11–12 min, and found no differences in terms of survival rate between the groups. But implantation and live birth rate (LBR) were lower in the 5–6 min exposure group compared with the three other groups. These preliminary data need to be confirmed by additional future studies. Finally, further epidemiological studies performed on a large-scale are necessary to evaluate the implications of the cryopreservation process and CPAs on the health and wellbeing of the offspring, not only at the time of delivery but also during later adult life.

**TABLE 1:** Minimal concentration required to vitrify for some permeating cryoprotectants at a pressure of 1 atmosphere according to Fahy and colleagues 1984. PG, propylene glycol; DMSO, dimethyl sulfoxide; EG, ethylene glycol; GLY, glycerol.

Cryoprotectants	Concentration required to vitrify %/volume
DMSO	49–50
PG	43.5
EG	55
GLY	65

## Indications for FET in ART practice

Recent societal modifications and the increasing desire and opportunity to preserve fertility for a variety of reasons, have raised the application of ART and have also increased the reasons for which ART is currently utilised. In this scenario, lately, embryo cryopreservation plays an active role in ART, and it is routinely and extensively applied (Table 2). In the past, embryo cryopreservation was adopted to store a surplus number of embryos following fresh ET, for infertile women undergoing ART. Nowadays, the storage of gametes (sperm and oocytes), embryos, and reproductive tissues (ovarian and testicular tissues) for use in ART is included in the field of fertility preservation. Thus, advancements in cryopreservation allow scientists to safely handle those cells and tissues, which represent a unique and valid option for cancer patients, who can cryo-store their reproductive tissues for future use once they have completed their cancer therapy (Somigliana et al., 2015; Sciorio and Anderson, 2020). Indeed, further application of this technology includes those women who lack the ability to produce their own eggs or who have a hereditary condition they wish to avoid passing on, who now have the option to receive donated embryos thanks to cryopreservation. Other interesting reasons why individuals adopt cryopreservation include age-related changes in gender or gender transitioning, preimplantation genetic testing (PGT), and ovarian hyperstimulation syndrome (OHSS) (Sciorio and Esteves, 2020). Indeed, PGT relies upon embryo cryopreservation which allows for the time interval between blastocyst biopsy and genetic analysis (Coates et al., 2017). Furthermore, vitrification is also very useful for other medical reasons such as severe endometriosis or elevated progesterone in the late follicular phase, which has been reported to have a negative impact on pregnancy outcomes; in such instances, it is recommended to cryopreserve all available embryos and perform a FET in a future cycle (Venetis et al., 2013; Santos-Ribeiro et al., 2014; Groenewoud et al., 2018). To prevent the risk of OHSS, a potentially life-threatening complication, fresh ET cannot always be performed (Kawwass and Kissin, 2015; Sciorio and Esteves, 2020). Finally, since multiple pregnancies are one of the most critical and avoidable problems in ART, culturing embryos until the blastocyst stage, and vitrifying every single good quality blastocyst for future use, represents a valid alternative to reduce the incidence of multiple pregnancies, while still maintaining high cumulative pregnancy rates. This approach has been reported in several studies by others (Liebermann and Tucker, 2006; Johnston et al., 2014; Sciorio et al., 2018; Sciorio et al., 2019; Liebermann, 2021; Liebermann et al., 2023).

**TABLE 2:** Main indication for human embryo cryopreservation in ART treatments.

Indication	Rationale for cryopreservation
**Preimplantation genetic testing**	Genetic assessment is facilitated by the opportunity to utilize cryopreservation to store embryos to be transferred in a future cycle, and to overcome the necessary time interval between blastocyst biopsy and genetic analysis (Coates et al., 2017)
**Risk of o**varian hyperstimulation syndrome **(OHSS)**	When fresh embryo transfer cannot be performed due to the risk of exacerbating OHSS should pregnancy occur, embryos might be cryopreserved and used in a future cycle (Kawwass et al., 2015; Sciorio and Esteves, 2020)
**Elective single embryo transfer (eSET)**	The cryopreservation of surplus embryos is considered a valid method to reduce the number of embryos transferred during a fresh cycle and thus minimize the risk of multiple pregnancy, and reduces the need for repeated stimulation cycles [Johnston et al., 2014; Sciorio et al., 2019; Liebermann, 2021]
**Preservation of fertility**	In women with a stable partner about to go through gonadotoxic/chemotherapy treatments for cancer, there may be time in which to undergo a cycle of IVF and have blastocysts cryopreserved [Somigliana et al., 2015; Sciorio and Anderson, 2020]
**Elevated progesterone or other conditions affecting endometrial receptivity, such as endometriosis, endometritis, hydrosalpinges, and fluid within the endometrial lumen**	Elevated progesterone in the late follicular phase has a negative impact on pregnancy outcomes. Or other conditions and medical pathology that might affect fertility (Venetis et al., 2013; Groenewoud et al., 2018)

## Application of double vitrification (re-cryopreservation)

In ART, sometimes it might be useful to perform a repeated cryopreservation event, in order to further increase the cumulative clinical pregnancy rate and reduce the risk of multiple pregnancies. Also, double vitrification-warming has been described to allow retesting of inconclusively diagnosed blastocysts in PGT, to circumvent limitations associated with national policies on embryo culture in certain countries, and in the case of donor vitrified-warmed oocytes that following fertilization are cultured to the blastocyst stage and re-vitrified for future use. A recent retrospective study by Hallamaa and collaborators, investigated a cohort of vitrified and slow-frozen embryos and reported no detrimental impact of double cryopreservation on clinical and neonatal outcomes (Hallamaa et al., 2021). Another study published by Makieva and colleagues has investigated this aspect, comparing the clinical pregnancy rate (CPR) and LBR following double vitrification-warming in those cycles where vitrification was performed first at the zygote stage and the second procedure occurred at the blastocyst stage in the absence of biopsy (Makieva et al., 2023). The authors in this retrospective analysis compared the pregnancy outcomes following single blastocyst transfers in embryos obtained after single vitrification-warming (n = 310) with those of double vitrification-warming (n = 97). Results showed a similar CPR (44.3% in double *versus* 42.3% in single vitrification) and LBR (30.9% in double *versus* 28.7% in single vitrification) between the two groups. Also, the miscarriage rate was comparable in the two groups (27.9% in double and 32.1% in single vitrification). A study by Shen and co-authors has investigated the perinatal outcomes of singletons born following events of embryo re-cryopreservation (Shen et al., 2023). This was a retrospective study, in which a total of 647 singleton live births after FET were analysed, of which 55 cases were once vitrified blastocysts, and 592 cases were twice vitrified blastocysts. Results showed comparable birthweights between the two groups (3,390.6 ± 601.5 g *versus* 3,412.8 ± 672.6 g, *p* > 0.05). Also, the percentage of preterm birth (20.4% *versus* 16.7%), low birthweight (3.7% *versus* 7.4%), macrosomia (11.1% *versus* 16.7%) and large for gestational age (LGA: 29.6% *versus* 22.2%) were not significantly different between the two groups. Following logistic regression analysis, the authors concluded that double vitrification-warming events did not impair perinatal outcomes (Shen et al., 2023). Re-cryopreservation has also been also investigated by Wang and colleagues in a systematic review and meta-analysis (Wang et al., 2023). The authors analysed 14 studies including 4,525 FETs, with 3,270 following single cryopreservation and 1,255 after double cryopreservation procedures. In contrast with the previously cited studies, Wang’s investigation reported that double vitrification is associated with a decreased LBR (OR, 0.67; 95% CI, 0.50–0.90) and an increased miscarriage rate (OR, 1.52; 95% CI, 1.16–1.98) when compared with single cryopreservation. No significant difference was found in neonatal outcomes. Therefore, since re-cryopreservation might impair embryo viability, clinical teams should maintain a cautious attitude toward double vitrification events during ART cycles.

## Efficiency of vitrification protocols

Unlike conventional slow freezing protocols, vitrification strategies enable plunging of the blastocyst into LN_2_ at −196°C after a significantly shorter exposure to CPAs (150 min *versus* 5–15 min). The transition from an aqueous solution into a glass-like solid during the cooling curve minimises significantly the possibility of ice formation during the process, and therefore any potential impact on blastocyst integrity, survival, and clinical outcomes (Nagy et al., 2020). Globally, zygotes and cleavage-stage embryos display comparable and consistent results with slow freezing, while at the blastocyst stage, the increasing amount of water contained within the blastocoel fluid could jeopardize the efficiency of the standard traditional cryopreservation method. The survival rate of cryopreserved embryos after warming represents a clear and immediate measure of efficiency for a cryopreservation procedure (Stehlik et al., 2005; Balaban et al., 2008; Nagy et al., 2009; Valojerdi et al., 2009; Cobo et al., 2013). Since the introduction of vitrification, IVF laboratories have benefited over recent decades from vitrification’s consistently high survival rate, simplicity, and reduced time, which has contributed to widespread implementation (Nagy et al., 2009; Wilding et al., 2010; Fasano et al., 2014; Debrock et al., 2015). So far, data has revealed a significant improvement in blastocyst post-warming cryo-survival rates (Sifer et al., 2012; Wang et al., 2012; Liu et al., 2013; Zhu et al., 2015; Summers et al., 2016). A meta-analysis published by Rienzi and colleagues, based on several randomized controlled trials (RCTs), supports vitrification as a superior approach to slow-freezing, not only in blastocysts but also in human oocytes and cleavage-stage embryos. Even though high variability was reported among laboratories, the implementation of vitrification in a regular facility led to an increase in embryo cryo-survival rate (from ∼60% to 78%–100%) along with increased CPR and LBR per embryo when compared to slow-freezing (Rienzi et al., 2017). Globally, this technique has significantly improved embryo post-warming survival rates, even though there is considerable variation (30%–93%). In general, the current efficiency of vitrification technology is so high that almost all vitrified blastocysts survive and preserve their implantation potential (Valojerdi, et al., 2009). The improved viability of vitrified-warmed blastocysts is directly translated into increased implantation and pregnancy rates, leading to equal or even higher results than those with fresh embryo replacement (Cobo et al., 2012; Ozgur et al., 2015; Wang et al., 2017). However, the specific procedure varies from laboratory to laboratory, contributing to significant variability. Though some attempts with automatic vitrification have been described by others (Roy et al., 2017; Arav et al., 2018), and will be subsequently discussed, vitrification remains a predominantly manual and highly operator dependent procedure.

## Vitrification protocols

**FIGURE 1:**
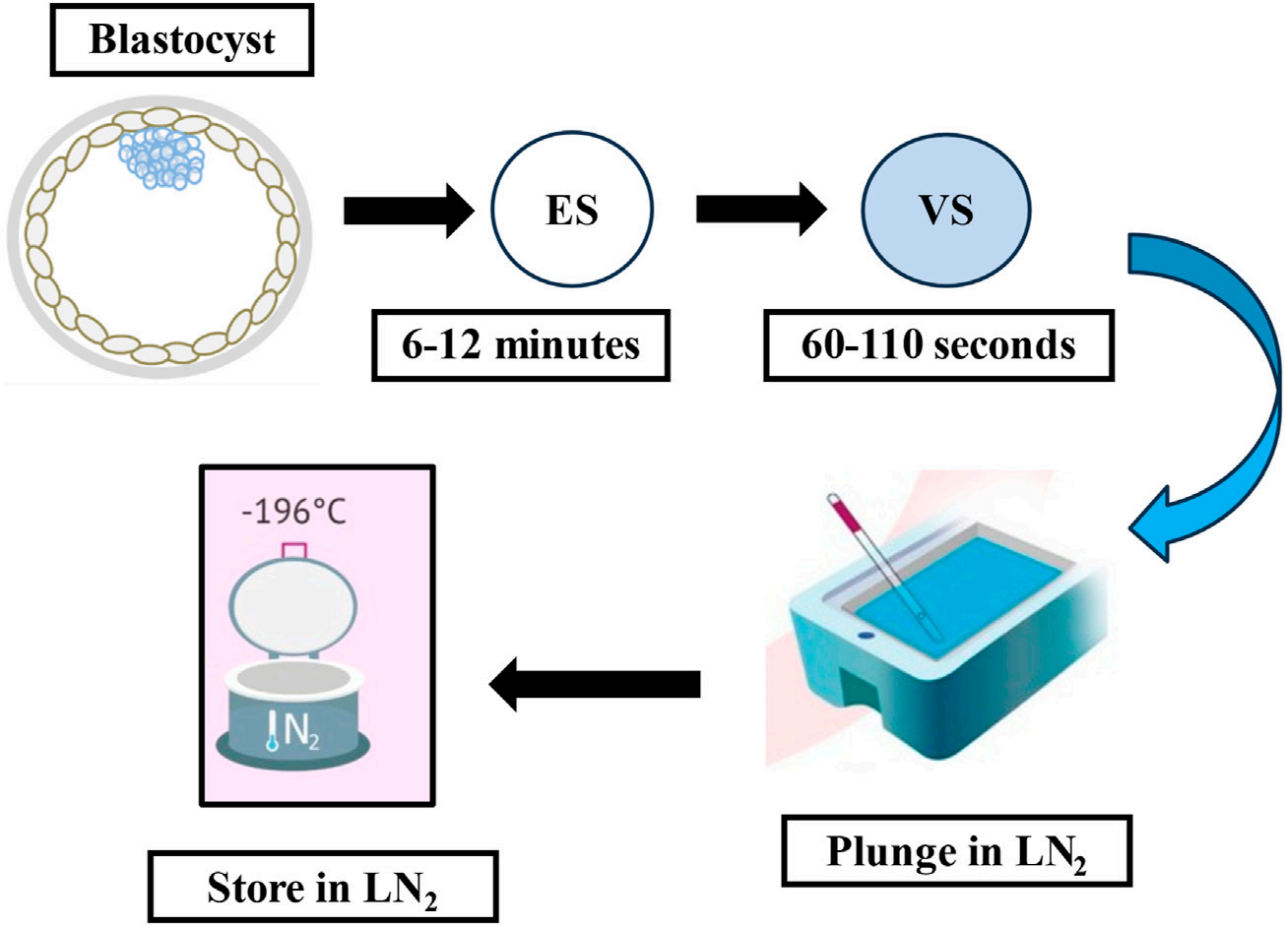
The flow-diagram illustrates the cryopreservation process using the vitrification method. ES: equilibration solution, VS: vitrification solution, LN_2_: liquid nitrogen.

**FIGURE 2:**
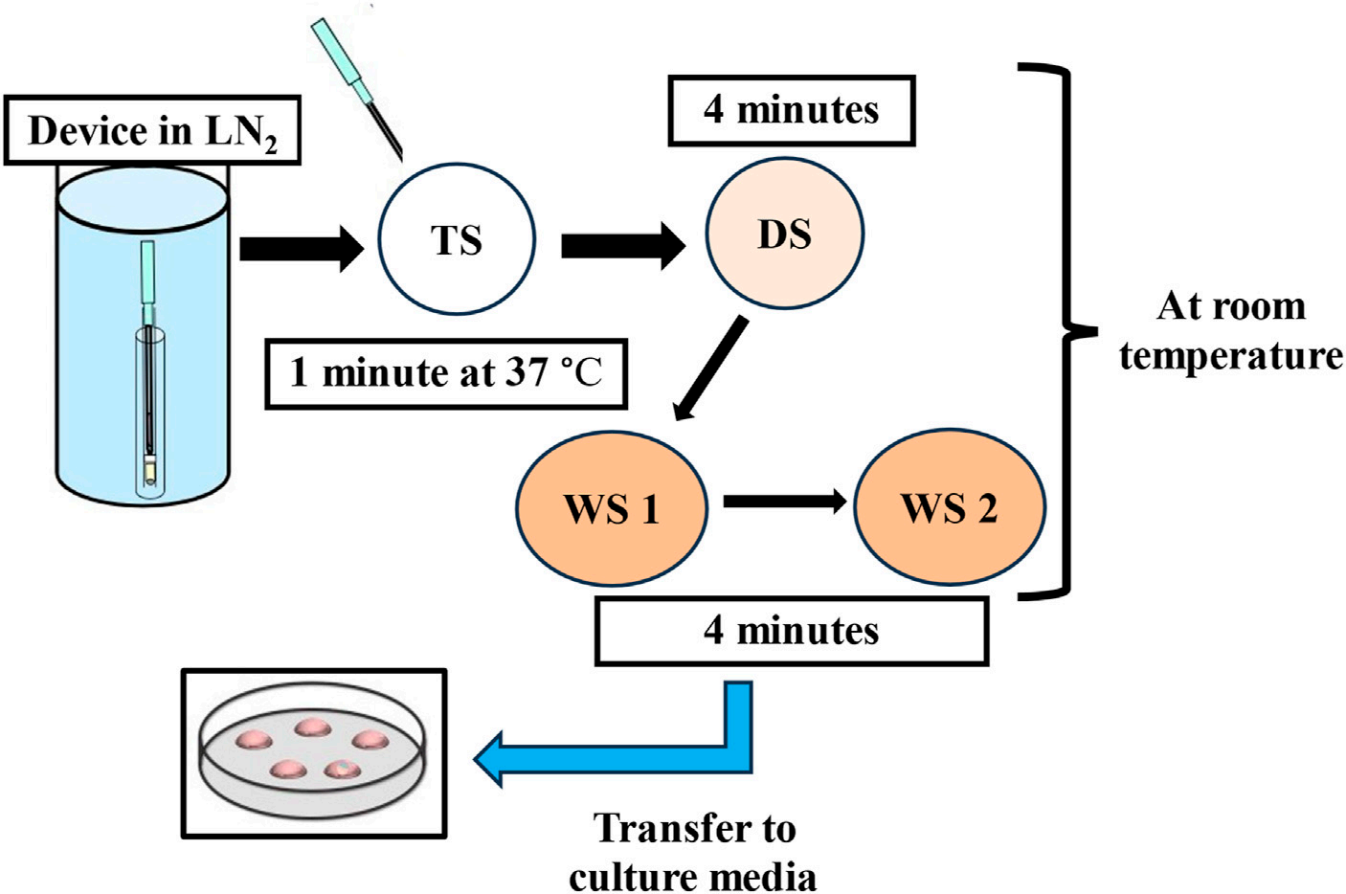
The flow-diagram shows the warming procedure for vitrified blastocysts. TS: thawing solution, DS: dilution solution, WS: washing solution, LN_2_: liquid nitrogen.

Every vitrification (also called non-equilibrium cryopreservation) protocol is based on the same principle: a short exposure to small volumes of highly concentrated solution containing CPAs and a very high rate of cooling/warming (>10,000°C/min) to prevent the formation of intercellular and intracellular ice crystals. A wide variety of commercial kits, including different cryoprotectant solutions, carrier tools and times of exposure have been described. Current approaches use a combination of different CPAs at lower concentrations to ameliorate the toxicity of one single CPA at an otherwise higher concentration (Rall et al., 1987; Ali and Shelton, 1993). Following the introduction of open-pulled straws such as the Cryotop, closed systems have been developed to avoid contact with LN_2_ and thereby provide a safer and more sterile alternative (Vajta et al., 2015). Most current devices share a common design that allows them to employ small volumes of vitrification solutions to achieve the highest rates of temperature change (Nagy et al., 2020). One of the most adopted vitrification protocols (the rest are identical or slightly modified) uses a minimum volume (≤1 μL) carrier device called the Cryotop together with a mixture of two permeating CPAs, 15% (2.7 M) EG and 15% (2.1 M) DMSO plus 0.5 M sucrose or trehalose as external CPAs (Figure 1) (Kuwayama et al., 2005). Previously, the embryo is maintained for 10–15 min in the equilibration medium (EG: 1.35 M + DMSO: 1.05 M). Based on an osmolality of approximately 280 mOsm/Kg in culture media, the exposure and removal of CPAs entails extreme osmolality changes during vitrification and warming procedures. The osmotic pressure shifts up to 2,700 mOsm/Kg in the equilibration solution and then is further raised to 5,600 mOsm/Kg when the embryo is briefly (1 min) transferred to the vitrification solution prior to being submerged in LN_2._ The warming procedure mainly involves a rapid dilution and reduction in osmolality from 5,600 to 1,280 mOsm/Kg in the warming solution (37°C), followed by two re-hydration stages at room temperature further reducing osmolality to 780 mOsm/Kg (3 min) and then 280 mOsm/Kg (5–6 min). Typically, the warming kit contains 1.0 M sucrose (thawing solution; TS), 0.5 M sucrose (dilution solution; DS), and HEPES buffered solution alone (washing solution, WS) which allows control over the speed of extracellular water diffusion and controls cell swelling during re-hydration (Figure 2). A cell’s membrane may be ruptured if it rehydrates too fast (Kuwayama et al., 2005). Finally, it is worth mentioning that during the vitrification-warming procedures the blastocyst is exposed to non-physiological oxygen tension, and the high concentration of oxygen might be correlated with increased levels of ROS and apoptotic events, with potential repercussions in the adult life of the conceived baby (Sendzikaite and Kelsey, 2019; Verheijen et al., 2019; Barberet et al., 2020; Chen et al., 2022). In that respect, the addition of antioxidants to vitrification media may be beneficial for embryo development, especially under oxidative stress. A study from Truong and Gardner, investigated the benefits of an antioxidant combination, specifically N-acetyl-L-cysteine, acetyl-L-carnitine, and α-lipoic acid, during vitrification of mouse embryos (Truong and Gardner, 2020). The study showed that mouse blastocysts vitrified with no antioxidants had significantly lower cell numbers (*p* < 0.001) and higher apoptotic cells (*p* < 0.05) compared to non-vitrified embryos. Addition of antioxidants during the vitrification and warming protocols was associated with a significant increase in inner cell mass (ICM) number (*p* < 0.001) and total cell number (*p* < 0.01), and an increase in outgrowth area (*p* < 0.05) compared to embryos vitrified without any antioxidants. This aspect might be further developed as it may present an intriguing avenue for enhancing protocols.

## Ultra-fast warming method

Despite the high efficiency of current protocols, over recent years alternative approaches have been proposed to improve results and optimize laboratory workflow. The main critical concerns in successful vitrification of the human embryo are the removal of cytotoxic CPAs and rehydration in serial osmotic solutions to prevent osmotic shock, to increase cryo-survival. Logic implies to quickly remove CPAs and allow water to gradually rehydrate the embryo to minimize damage during the vitrified-warming process. This feature is normally achieved through multi-step warming strategies, that involve moving the blastocyst through a series of solutions of decreasing osmolality to remove the CPAs, and have shown good outcomes, though the procedure is certainly time-demanding (10–15 min) and is a laborious routine in the IVF laboratory. Thus, reducing the amount of time required to rehydrate the embryo has been proposed as an alternative ultra-fast warming approach (Manns et al., 2021; Manns et al., 2022; Taylor et al., 2022). Ultra-fast warming involves neither dilution nor washing and only requires plunging the embryo into the TS for 1 min at 37°C before transfer into culture media. So far, the 1-min one-step rehydration approach has shown consistently high survival rates and, more importantly, comparable implantation and pregnancy rates (Manne et al., 2021; Manns et al., 2022; Taylor et al., 2022; Liebermann et al., 2023). In a study by Gallardo and colleagues, the authors advocate that during the vitrification-warming protocol, reducing the time and the duration of the procedure would be desirable to improve the workflow in the IVF setting and also reduces the duration of exposure to suboptimal temperature, osmolality, a high concentration of oxygen, and potential toxicity of CPAs. In this work they investigated a short, rehydration-based protocol, in which the warming was performed with only 1 minute exposure in TS, compared to a standard protocol, using discarded human oocytes and abnormally fertilized zygotes donated for research (Gallardo et al., 2019). With human oocytes and abnormal (3PN) zygotes exposed to this experimental vitrification protocol with 1 minute rehydration, survival rates were 30/30 (oocytes) and 27/27 (zygotes), which was comparable to the standard warming procedure. Of those 27 survived abnormal zygotes (3 PN), 24 managed to cleave in culture after 24 h. A study by Liebermann and co-workers, which is the largest published study so far, showed that not only is fast warming quicker and simpler than the multi-step protocol, but also the results are quite promising (Liebermann et al., 2023). They retrospectively analyzed 3,439 FETs, and they compared clinical outcomes between the one-step rehydration protocol and a control group, which was the standard multi-step rehydration protocol. Results showed the same survival rates for the two arms (99.5%). The CPR was 63.0% in the one-step warming protocol, which was comparable to 59.9% in the multi-step rehydration protocol. A significant increase was observed in the ongoing pregnancy rate (60.4% in the one-step rehydration *versus* 55.4% in the multi-step rehydration groups, *p* = 0.011) and implantation rate (63.6% in one-step *versus* 57.0% in the control group, *p* = 0.0005), with significantly lower spontaneous miscarriage rates (4% in one-step *versus* 7.6% in the control arm, *p* = 0.0001). Shortening the time that embryos are out of the incubator during the one-step warming protocol may contribute to these higher outcomes. Globally, in addition to the significant time saving (from 10–15 to 1 min of bench time for the embryologist), eliminating DS-WS steps results in identical survival rates and, above all, does not negatively impact pregnancy or implantation rates. In that respect, an extreme approach has been recently proposed by Chan and co-authors, who have validated ultra-fast warming of vitrified human blastocysts by immerging them directly into regular embryo culture media devoid of any CPAs (Chan et al., 2024). The study was divided into pre-clinical and clinical phases. In the first technical stage, 63 donated human blastocysts were warmed directly in five different types of culture media. The blastocysts were immerged in pre-warmed medium at 37°C (pre-equilibrated overnight) and incubated for 1 minute at a heat-top workstation before being transferred to a time-lapse incubator for further observation. They reported a survival rate of 100%, assessed with time-lapse technology and observation of re-expansion following warming. The second stage was the clinical phase, which included a cohort of 96 patients who were scheduled for FET, 20 of which were subjected to direct warming in culture media: 19 of these were single ET and one patient was for double ET. All warmed blastocysts survived in both groups (one-step and standard warming procedures). The implantation rate in the direct warming arm was significantly higher [61.9% (13 sacs/21 blastocysts)] compared to the standard warming procedure [37.2% (29/78)]. Though the LBR was higher in the direct warming group [45% (9 live births/20 FETs)] compared to the standard group [36.8% (28 live births/78 FETs)], this difference was non statistically significant. The direct thawing procedure in embryo culture medium reduced embryology time and significantly reduced the cost per FET performed. The saving was estimated by the authors to be around 90% per FET.

## Blastocyst shrinkage before vitrification, and assisted hatching post-warming

Cryopreservation of human blastocysts, using the vitrification approach, should consistently avoid intracellular ice-crystal formation compared to the traditional slow-freezing method; however, the large fluid-filled cavity in expanded blastocysts may inhibit sufficient permeation of CPAs inside the blastocoel, and might be responsible for ice crystal formation which can induce cell death. Applying artificial shrinkage (AS), thus reducing the volume of the blastocoel might increase the survival rate at warming and pregnancy outcomes following FET. Several authors have described different methods to induce AS, such as creating a hole in the trophectoderm layer, either by puncturing it with a needle (Son et al., 2003), by repeated micropipetting of the blastocyst (Hiraoka et al., 2004) or by laser pulse (Mukaida et al., 2006). Studies by different authors using these methods, have reported being able to obtain immediate collapse of the blastocoelic cavity just before vitrification, resulting in a positive effect on survival after warming (Vanderzwalmen et al., 2003; Mukaida et al., 2006; Levi-Setti et al., 2016; Wang et al., 2017; Sciorio et al., 2018). Therefore, use of AS by a laser pulse or any other method described earlier, might increase the diffusion of cryoprotectants into the embryo, and thus, the embryo’s exposure to the equilibration solution can be reduced to obtain an efficient vitrification process (Sciorio et al., 2019). Fully expanded blastocysts include a high amount of fluid in the blastocoel cavity, which during the process of vitrification can produce ice crystals, therefore those blastocysts might benefit from AS to augment vitrification efficiency. Using a laser pulse, those expanded blastocysts can be easily collapsed, lose fluid in a short time and be converted into a morula-like stage. Laser technology is simple, accurate and effective, and has been applied in different fields, including ART for more than 30 years. A laser pulse at a minimal setting, orientated at the gap junction between two trophectoderm cells, away from the ICM, can be applied to induce AS just a few minutes before the vitrification starts (Sciorio et al., 2018), to improve cryo-survival. This approach has been amply reported by others (Vanderzwalmen et al., 2003; Mukaida et al., 2006; Van Landuyt et al., 2015; Levi-Setti et al., 2016; Wang et al., 2017; Sciorio et al., 2019; Kovačič et al., 2022). The application of a laser can also be used to induce zona drilling, in a procedure generally known as assisted hatching (AH) in both fresh and FET; this aspect has been investigated by several authors with controversial results. Retrospective trials have reported no benefit of the procedure (Graham et al., 2000; Dayal et al., 2006), except for some specific groups of patients: such as advanced maternal age (Meldrum et al., 1998), poor prognosis patients, or those with previous failed IVF cycles (Cohen et al., 1992; Schoolcraft et al., 1994; Grace et al., 2007). Sifer and collaborators (Sifer et al., 2006), in a prospective randomized study of cryopreserved embryos at the cleavage stage, reported no improvement in pregnancy outcomes: a similar implantation and CPR was observed between the AH and control groups. In contrast, a prospective blinded randomized study (Gabrielsen et al., 2004) performed on FET at the cleavage stage found an increased implantation rate in the AH group compared with the control group (11.4% *versus* 5.8%; *p* < 0.005). Also, Vanderzwalmen and collaborators found encouraging results following AH; they analysed 281 blastocysts after vitrification and warming, concluding that artificial opening of the ZP significantly increased the percentage of implantation and pregnancy rates (Vanderzwalmen et al., 2003). Finally, two recent studies again reported divergent findings. Wei and colleagues analysed 3,535 FETs, out of which 2,297 were non-AH cycles and in 1,238 laser AH was applied (Wei et al., 2023). Their results found a higher LBR in the AH group compared to the non-AH group (34.9% *versus* 31.4%, *p* = 0.024). Furthermore, the laser AH group showed a reduction in pregnancy loss and ectopic pregnancy rates, but those variations were not statistically significant (*p* = 0.078, *p* = 0.063 respectively). Opposite results were found by Alteri and collaborators in a comparative RCT performed in two centres (Alteri et al., 2024). The investigation enrolled 698 participants, which were randomized as follows: 352 patients were assigned to the AH group and the remaining 346 to the control arm. AH was applied to remove approximately one-third of the zona pellucida. The primary outcome of the study was LBR; and similar results were reported [105 (29.8%) in AH arm, *versus* 101 (29.2%) in the control group]. Secondary end-points included CPR, miscarriage, multiple pregnancies, and the authors were unable to find any clinical scenario that could benefit from AH in thawed blastocysts. Overall, based on the findings presented above, it seems that currently there is not enough evidence showing a clear benefit of AH in FET, in terms of LBR and pregnancy outcomes.

## Automated vitrification

Over the last decade, we have witnessed an incremental application of automated systems to perform mainstream laboratory procedures with the goal of increased standardization in methodologies and results, as well as decreasing the manual workload. Along those lines, application of automated vitrification platforms may help to standardize the procedure, lower the variation in performance between operators and cut down the amount of time-consuming manual work in the embryology laboratory. The first attempt of semi-automated vitrification was reported by Roy and colleagues; they found a decrease in time spent with similar laboratory outcomes to both human and mouse blastocysts vitrified using a manual method (Roy et al., 2014; Roy et al., 2017; Arav et al., 2018). The authors used an automated platform, the so called “Gavi^®^ system”, which was developed by a team of embryologists, scientists, and engineers at Genea in Sydney, in collaboration with Planet Innovation (Melbourne, Australia). The Gavi^®^ system can execute automated vitrification using a closed system of up to four embryos simultaneously. This system is a semi-automated machine for vitrification capable of monitoring critical features including temperature, volume, concentration, and exposure time to CPAs during the vitrification procedure. The system includes an instrument that performs fluid exchange using a robotic liquid handling unit with individual pipettes, has a heat-sealing unit, and includes a LN_2_ bucket. Further details of the Gavi^®^ system have been described by others (Roy et al., 2014). Using the Gavi^®^ system, the authors found equivalent *in-vitro* outcomes with mouse embryos to that of Cryotop controls. They vitrified mouse blastocysts with both the Gavi^®^ system (n = 176) and the manual Cryotop method (n = 172) and achieved a 99% recovery rate, of which 54% and 50%, respectively, progressed to fully hatched blastocysts 48 h after warming. Though the number treated was lower, human blastocysts vitrified with the Gavi^®^ system (n = 23) or with Cryotop controls (n = 13), resulted in a 100% recovery for both groups, of which 17% and 15%, respectively, progressed to fully hatched blastocysts 48 h after warming (Roy et al., 2014). Dal Canto and colleagues have recently reported the first two cases of ongoing pregnancy in Europe, following blastocyst vitrification/warming using the Gavi^®^ vitrification system (Dal Canto et al., 2019). Another study on the application of the Gavi^®^ system was published by Miwa and co-workers (Miwa et al., 2020). The authors retrospectively compared the survival rate, and clinical and perinatal outcomes following vitrified-warmed blastocyst transfer between Gavi^®^ (398 cases) and the Cryotop (208 cases). They found similar survival rates [Cryotop: 98.6% (208/211) *versus* Gavi^®^: 99.3% (398/401)], pregnancy rates [Cryotop: 34.3% (72/208) *versus* Gavi^®^: 33.4% (133/398)], and comparable miscarriage rates between the two groups [Cryotop: 22.2% (16/72) *versus* Gavi^®^: 24.8% (33/133)]. From those studies, it can be concluded that Gavi^®^ semi-automated vitrification can be considered as an alternative vitrification procedure in ART and might be introduced into routine laboratory practice, especially in a busy IVF program (Dal Canto et al., 2019; Miwa et al., 2020). Another semi-automated vitrification device, recently produced by a company located in China (Biorocks Company Limited), is able to incorporate the CPAs and delivery to the cell using the form of hydrogel. This device is able to achieve a cooling rate of 31,900°C/min, and warming rates of 24,700°C/min. This innovative device has been described in a recent publication by Wang and co-workers (Wang et al., 2023). To assess the efficacy of this device (Biorocks vitrification system) the authors used mouse oocytes and embryos, and poor quality human day 6 blastocysts (grade CC according to Gardner and Schoolcraft, 1999), and obtained outcomes equivalent to the manual Cryotop method. They reported a survival rate of 98% for mouse oocytes with the Biorocks system (n = 46) and 95% for the Cryotop (n = 39), of which 46% and 41%, respectively, progressed to blastocysts on day 5 after IVF. Regarding the human blastocysts (day-6 grade CC) processed with the Biorocks device (n = 39), a re-expansion rate of 92% was observed within 2 h post warming, compared to 90% obtained with the Cryotop (n = 30). However, these are the very early days of automated vitrification, and there might be extensive opportunity for improvement. Therefore, we believe that further and larger well-designed studies are required to evaluate its impact upon CPR and LBR.

## Vitrification: neonatal outcomes, safety, and potential adverse obstetric complications

Recent data show that an increasing percentage, between 30% and 40%, of children born following ART cycles worldwide result from cryopreservation practices, from both cleavage and blastocyst stages (Roy et al., 2014; Kupka et al., 2016). Furthermore, concerns associated with the health of ART-conceived babies, as well as from FETs, have been discussed for several years, and have been the object of many investigations by different research groups. Currently, not much is known about the safety of the technique especially on obstetric complications in long-term follow-up. With the increasing use of vitrification, other risks seem to be highlighted, especially those associated with high, potentially toxic concentrations of CPAs, compared to what was used in the slow-freezing procedure. One of the concerns investigated by several authors has been the potential detrimental effect that the duration of storage time may have on vitrified human embryos, especially when some studies found an association between cryo-storage length and decreased clinical results. A study by Cobo and co-workers evaluated this concern in a retrospective study including 58,001 vitrified/warmed day-5 blastocyst transfers. The storage time ranged from ≤1.8 months to ≥34.81 months. Their results found that blastocysts did not show statistical differences across the categories of storage time; and no association was found between storage time and clinical outcome (Cobo et al., 2024). Regarding the perinatal outcome of children born following vitrification, observational studies have revealed some impairments, such as an increased risk of placental problems, pregnancy induced hypertension, and pre-eclampsia following FET (Sazonova et al., 2012; Opdahl et al., 2015; Barsky et al., 2016; Jeve et al., 2016). A systematic review and meta-analysis published by Jeve and collaborators, including 81,752 cycles compared the obstetric outcomes among FET and fresh transfer (Jeve et al., 2016). The authors found that the risk of developing hypertensive disorders in pregnancy was significantly higher following vitrification. Other outcomes including SGA, caesarean section, and preterm delivery, as well as hypertension and pre-eclampsia were all significantly higher in pregnancies obtained after vitrification (Sazonova et al., 2012; Opdahl et al., 2015; Barsky et al., 2016). However, a multicentre RCT analysing 2,157 women found no significant differences in pre-eclampsia or hypertensive disorders, as well as other obstetrical and neonatal complications between the two groups (FET and fresh ET) (Shi et al., 2018). Also, a study by Takahashi and co-authors analysed 1,129 vitrified blastocysts and showed no differences in obstetric outcomes for babies born after vitrified blastocyst transfers compared to those children born following fresh transfers. However, there was a preterm birth rate of 18.5% compared to 12.4% in the fresh group (Takahashi et al., 2005). The debate is ongoing since several authors have reported perinatal and neonatal outcomes after FETs comparable to those following fresh ET (Devine et al., 2015; Ainsworth et al., 2019; Hwang et al., 2019; Maris et al., 2019). Furthermore, FET has been correlated with similar rates of congenital malformations when compared to fresh ET (Belva et al., 2016). A meta-analysis by Maheshwari and collaborators presents novel interesting evidence concerning divergences in terms of neonatal outcomes arising from fresh ET or FET (Maheshwari et al., 2018). Their analysis found that singleton children born from FET cycles were associated with a lower risk of preterm delivery or having low birthweight and SGA compared to those conceived following fresh ET, but a higher risk of high birthweight, LGA and, above all, their mothers faced an increased risk of hypertensive disorders during pregnancy. The authors found no difference in the risk of congenital anomalies and perinatal mortality, or admission to the neonatal intensive care unit between the two groups. However, an increased birthweight in ART babies conceived after FET has also been reported by large epidemiological studies in the UK (Maheshwari et al., 2016), and by several studies from Northern Europe (Pelkonen and Koivunen, 2010; Pinborg et al., 2013; Pinborg et al., 2014). In that regard, a study by Terho and co-workers (Terho et al., 2024), reported data extracted from the Finnish register from 1995 to 2006, and compared the singletons born following FET (n = 1,825), fresh ET (n = 2,933) and natural conception (n = 31,136). They found that adolescent boys (age 7–18 years), born following FET have a higher mean proportion and increased odds of overweight compared to those born after fresh ET. The FET boys had a higher mean proportion of overweight compared to fresh ET (28% *versus* 22%, *p* < 0.001) and compared to natural conception (28% *versus* 26%, *p* = 0.014). However, it is worth mentioning that it is very difficult to differentiate between whether the FET protocol itself is responsible for the higher birthweight or if other features might contribute such as the use of CPAs or mode of endometrial preparation. Along those lines, no difference in birthweight was seen by Shi and co-authors when embryos were transferred in a natural cycle, leading one to consider that endometrial preparation might play a crucial relevant role in that regard (Shi et al., 2018). Similar results were also found in a systematic review and meta-analysis performed by Zaat and colleagues (Zaat et al., 2023) including 1,546 studies in which FETs were compared between natural cycles (n = 56,445) and artificial cycles (n = 57,231). The authors reported a decreased risk of adverse obstetric and neonatal outcomes when a natural cycle was adopted compared with artificial treatments. An additional study by Rosalik and collaborators (Rosalik et al., 2021) also showed that programmed FET cycles resulted in a higher foetal weight, as well as higher risk for macrosomia and LGA when compared with natural FET cycles. Also, a Nordic register study by Terho and co-workers found that singletons born after FET are heavier and there is a higher risk of LGA compared to fresh ET (Terho et al., 2021). A critical aspect worth mentioning is the potential risk that the vitrification procedure might induce on epigenetic dysfunction and, by doing so, impair embryonic gene expression and imprinting. Potential consequences might include alterations in placenta and foetus formation, and induced modifications in growth patterns and metabolic parameters, potentially resulting in adult life diseases (De Baun et al., 2003; Cassidy et al., 2012; Azzi et al., 2014; Xu and Xie, 2018). Indeed, epigenetic regulation and imprinted genes play critical roles in cell growth and differentiation, and it is important to avoid any alteration, especially during the first few days of embryo development when embryos are cultured in the embryology laboratory, that might otherwise generate disorders in the offspring (De Baun et al., 2003; Mabb et al., 2011; Cassidy et al., 2012; Fleming et al., 2018). However, only limited evidence is currently available in humans and those investigations seem to suggest that imprinted genes and DNA methylation are not significantly altered following vitrification (Liu et al., 2017) (Table 3). An example is the study from De Munck and collaborators, who reported no significant change in the overall DNA methylation level of *in-vitro* cultured 8-cell embryos derived from vitrified/warmed oocytes (De Munck et al., 2015). However, in contrast, Huo and co-authors have studied a total of 1,987 genes and found different expression following oocyte vitrification/warming compared with fresh oocytes and reported that 82% of these genes were downregulated, while 18% were upregulated (Huo et al., 2021). Most of the genes investigated were involved in several critical biological processes or were cell cycle related, such as *NCAPD2, TUBGCP5* and *TUBB4*. Also, other aberrant gene expression after vitrification was found in genes whose activities were correlated with oogenesis, cellular response to heat, microtubule-based processes, methylation, ubiquinone biosynthetic processes, chromosome migration, DNA repair, as well as ATP production and metabolic processes, which are overall biological processes correlated with oocyte quality and viability (Stigliani et al., 2015). Along those lines, a registry-based cohort study using data from the four Nordic countries, Denmark, Finland, Norway, and Sweden, performed by Sargisian and co-authors (Sargisian et al., 2022) aimed to analyse whether children born after ART and specifically after FET, are at higher risk of childhood cancer compared to fresh ET or natural conception. The study included around eight million children, with 171,774 babies born after use of ART and 7.772,474 children born after natural conception. After adjustment performed for sex, plurality, year of birth, country of birth, maternal age at birth, and parity, the authors found that individuals born after FET had a higher risk of cancer (48 cases) when compared to natural conception or fresh ET. There were higher risks of epithelial tumors and melanoma after any ART method, and of leukemia after FET. However, the authors concluded that those results should be interpreted with high caution, considering the limited number of children with cancer (n = 48) (Sargisian et al., 2022). To conclude, considering the fast spread of vitrification procedures in modern ART cycles, further follow-up studies are urgently required to clarify and better understand the functional processes that are responsible for complications associated with FET and any potential epigenetic risks associated with vitrification (Hiura et al., 2017; Marjonen et al., 2018; Osman et al., 2018; Verheijen et al., 2019). Evidence already exists in humans showing that programmed FET cycles might have complications and adverse obstetric outcomes compared to natural conception or to FET in natural cycles. However, the epigenetic mechanisms responsible for those observed alterations remains generally unknown and currently are limited and marginally understood in humans. Further investigations are needed on the use of CPAs and vitrification, and large registry studies are essential to evaluate vitrification/warming procedures in humans, including neonatal outcomes and any potential long-term diseases.

**TABLE 3:** Summary of human studies showing the effects of vitrification on DNA methylation and histone modification. GV; oocyte at germinal vesicle stage, MII; oocyte at metaphase II stage, IVM; *in-vitro* maturation, 5hmC; 5-hydroxymethylCytosine, 5mC; 5-methylCytosine. DMR; differentially methylated regions.

Study [ref]	Materials Human or animal	Embryos analyzed (n)	Technology of assessment	Studied Sequences or genes	Main findings
De Munck et al. (2015)	**(Human)** Mature (MII) donated oocytes	31 embryos (Day-3) from 17 fresh oocytes and 14 after Vitrification	Immunofluorescence (5mC, 5hmC)	Global Analysis	No differences in fluorescence intensities between embryos from fresh and vitrified oocytes
Al-Khtib et al. (2011)	**(Human)** GV oocytes donated for research and IVM to MII	77 MII after IVM from 184 vitrified GV stage, and 85 MII from 120 fresh GV	Pyrosequencing	Methylation profile of H19 and KCNQ1OT1H19DMR and KvDMR1	Oocyte vitrification at the GV stage does not affect the methylation profiles of H19-DMR and KvDMR1
Liu et al. (2017)	**(Human)** Vitrified mature (MII) oocytes and MII from GV matured *in-vitro*	56 *in-vivo* MII, 106 MII from GV matured *in-vitro*, 122 MII from vitrified GV	Immunofluorescence (5mC)	Global analysis	No significant difference in fluorescence intensities between the groups
Barberet et al. (2020)	**(Human)** Placenta	Human placenta	Pyrosequencing and q-PCR	H19, IGF2, KCNQ1OT1 SNURF	The placental DNA methylation levels of H19/IGF2 were lower in the fresh embryo transfer group than in the control (H19/IGF2-seq1) and frozen embryo transfer (H19/IGF2-seq2) groups
Yao et al. (2020)	**(Human)** Placenta	Human placenta obtained from vitrified embryos	q-PCR, Western blot and pyrosequencing	SNRPN	The expression level of SNRPN increased after vitrification
Mani et al. (2022)	**(Human and Mouse)** Placenta	Human placenta following frozen and fresh ET. Mouse placenta from fresh and frozen ET	850K Infinium MethylationEPIC BeadChip array	Infinium Mouse Methylation BeadChip array	Human and mouse placentae were significantly hypermethylated after frozen ET compared with fresh

## Conclusive remarks

In the last few years, we have witnessed a consistent improvement in cryopreservation techniques. The vitrification method, considered a real breakthrough in ART, has almost replaced the traditional slow-freezing method, and has induced an absolute change in how physicians manage and handle IVF treatment. Indeed, there is enough published evidence showing that following FET, the implantation and pregnancy outcomes are comparable to those obtained from fresh ET in infertility patients undergoing ART and, therefore, FET has been perceived as a valid alternative to fresh ET. Thus, the widespread use of FET has found several and compelling applications, including when performing embryo biopsy for genetic testing or fertility preservation in cancer patients. Importantly, blastocyst vitrification represents a valid tool when introducing elective single ET, without compromising the pregnancy rate. Nowadays, extended culture until the blastocyst stage is more commonplace, allowing the selection of more viable embryos, and lowering the number of embryos to replace, especially when analysing the cumulative pregnancy rate from a single oocyte recovery. In addition, the higher number of cells within the blastocyst better compensates for any cryoinjuries, with considerable viability and faster recovery at the warming stage, resulting in a greater potential to implant. Finally, the safety aspects of cryopreservation to both women and their offspring necessitate further and long-term assessment. The use of high concentrations of CPAs might be associated with changes in cell biology mechanisms and asperity to promote alterations in the epigenetic landscape. Indeed, it is critical to further investigate these potentially negative long-term consequences that might be transmitted to later generations. However, despite the important role of such epigenetic mechanisms in cell-fate decisions, the number of reports available on the impact of CPAs and cryopreservation procedures on epigenetic mechanisms in the human is limited and remain conflicting. To conclude, blastocyst vitrification in ART has largely modified the application of ART, and its practice evolves as scientists learn more about ways to improve protocols and their application to specific groups of patients undergoing ART.
